# 3-Phenyl-2-(pyrrolidin-1-yl)-5,6-dihydro-8*H*-thio­pyrano[4′,3′:4,5]thieno[2,3-*d*]pyrimidin-4(3*H*)-one

**DOI:** 10.1107/S1600536810032794

**Published:** 2010-08-25

**Authors:** Shuang-Ming Meng, Hai Xie, Yue-Qin Fan, Bu-qin Jing, Yong Guo

**Affiliations:** a College of Chemistry and Chemical Engineering, Shanxi Datong University, Datong, Shanxi 037009, People’s Republic of China

## Abstract

In the title compound, C_19_H_19_N_3_OS_2_, the thio­pyran ring adopts a twist-chair conformation and the pyrimidinone unit is essentially planar, with a mean deviation of 0.0497 Å. The thio­phene ring is essentially planar with a maximum deviation of 0.024 (2) Å, while the pyrrolidine ring exhibits an envelope conformation. The pyrimidinone and thio­phene rings are almost coplanar, forming a dihedral angle of 6.31 (15)°, while the dihedral angle between the mean planes of the phenyl ring and the pyrimidinone ring is 68.13 (10)°. In the crystal structure, adjacent mol­ecules are linked by C—H⋯O hydrogen bonds, forming a two-dimensional network in the *ac* plane.

## Related literature

For the applications of pyrimidine derivatives as pesticides and pharmaceutical agents, see: Condon *et al.* (1993 ▶) and as anti­viral agents, see; Gilchrist (1997 ▶). For a related structure, see: Xie *et al.* (2008 ▶).
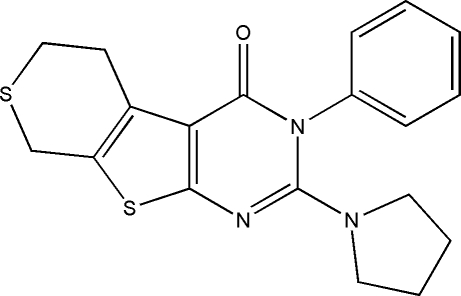


## Experimental

### 

#### Crystal data

C_19_H_19_N_3_OS_2_
*M* *_r_* = 369.49
Triclinic, 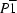

*a* = 8.1484 (8) Å
*b* = 9.3455 (9) Å
*c* = 12.1834 (12) Å
α = 73.668 (1)°
β = 88.629 (1)°
γ = 79.568 (1)°
*V* = 875.26 (15) Å^3^
*Z* = 2
Mo *K*α radiation
μ = 0.32 mm^−1^
*T* = 298 K
0.30 × 0.23 × 0.18 mm

#### Data collection

Bruker SMART CCD area-detector diffractometer
Absorption correction: multi-scan (*SADABS*; Sheldrick, 2000 ▶) *T*_min_ = 0.911, *T*_max_ = 0.945
4828 measured reflections
3349 independent reflections
2709 reflections with *I* > 2σ(*I*)
*R*_int_ = 0.016

#### Refinement

*R*[*F* ^2^ > 2σ(*F* ^2^)] = 0.043
*wR*(*F* ^2^) = 0.119
*S* = 1.06
3349 reflections
226 parameters
H-atom parameters constrained
Δρ_max_ = 0.50 e Å^−3^
Δρ_min_ = −0.44 e Å^−3^

### 

Data collection: *SMART* (Bruker, 2000 ▶); cell refinement: *SAINT* (Bruker, 2000 ▶); data reduction: *SAINT*; program(s) used to solve structure: *SHELXS97* (Sheldrick, 2008 ▶); program(s) used to refine structure: *SHELXL97* (Sheldrick, 2008 ▶); molecular graphics: *SHELXTL/PC* (Sheldrick, 2008 ▶); software used to prepare material for publication: *publCIF* (Westrip, 2010 ▶).

## Supplementary Material

Crystal structure: contains datablocks I, global. DOI: 10.1107/S1600536810032794/pv2315sup1.cif

Structure factors: contains datablocks I. DOI: 10.1107/S1600536810032794/pv2315Isup2.hkl

Additional supplementary materials:  crystallographic information; 3D view; checkCIF report

## Figures and Tables

**Table 1:** Hydrogen-bond geometry (Å, °)

*D*—H⋯*A*	*D*—H	H⋯*A*	*D*⋯*A*	*D*—H⋯*A*
C11—H11*B*⋯O1^i^	0.97	2.58	3.434 (3)	148
C1—H1*A*⋯O1^ii^	0.97	2.48	3.287 (3)	140

Symmetry codes: (i) 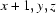; (ii) 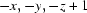.

**Fig. 1.**
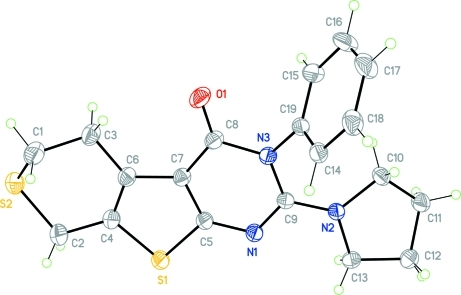
The structure of (I), with displacement ellipsoids drawn at the 30% probability level for the non-H atoms.

**Fig. 2.**
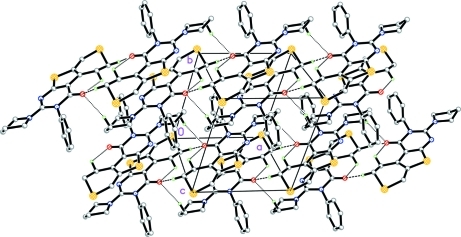
The H-bonds network of the title compound(I), dotted lines show C—H···O bonds.

## supplementary crystallographic information

### Comment

Pyrimidine derivatives are very important molecules in biology and have many application in the areas of pesticide and pharmaceutical agents (Condon *et al.*, 1993). Pyrimidine derivatives have also been developed as antiviral agents, such as AZT (azidothymidine), which is the most widely used anti-AIDS drug (Gilchrist, 1997). In order to discover further biologically active pyrimidine compounds, the title compound was synthesized, and its crystal structure was determined which is presented in this paper.

In the title compound (Fig. 1) the tetrahydropyran ring adopts a twist chair conformation with S2 and C1 lying 0.451 (5) and 0.474 (5) Å, respectively, on the opposite sides of the plane formed by the rest of the ring atoms. The pyridinone unit is essentially planar with a mean deviation of 0.0497 Å; the maximum deviation of any atom from the plane is 0.0432 (14) Å for N3. The pyrimidone and the thiophene rings are almost coplanar with the dihedral angle 6.31 (15)° and the dihedral angle between the mean-planes of the phenyl ring and the pyridinone ring is 68.13 (10)°. The thiophene ring is essentially planar (max. dev. 0.024 (2) Å for C4) while the tetrahydropyrrole exhibits a C12-envelop conformation with C12 lying 0.568 (4) Å out of the plane formed by the rest of the atoms in the ring.

The bond distances and bond angles in the title compound are comparable to the corresponding distances and angles reported for a closely related compound (Xie *et al.*, 2008). In the crystal structure, adjacent molecules are linked by C–H···O hydrogen bonding interactions (Tab. 1), and form a two-dimensional network (Fig. 2).

### Experimental

To a solution of iminophosphorane (2.0 mmol) in absolute anhydrous dichloromethane (10.0 ml), isocyanatobenzene (2.0 mmol) was added under nitrogen atmosphere at room temperature. After the reaction mixture was stirred for 3.0 h at room temperature, the iminophosphorane had disappeared (TLC monitored). The solvent was removed under reduced pressure and EtOH/petroleum ether (1:3, 15.0 ml) was added to precipitate triphenylphosphine oxide. Removal of the solvent gave carbodiimides, which were used directly without further purification. To a solution of carbodiimides in dichloromethane (10.0 ml) was added pyrrolidine (2.0 mmol). The reaction mixture was left unstirred for 6 h. The solvent was removed and anhydrous EtOH (10.0 ml) with several drops of EtONa was added. The mixture was stirred for 6–12 h at room temperature. The solution was condensed, workup and chromatography (hexane/AcOEt) gave the expected cyclic compounds in good yield. The crystals of the title compound were grown mother liquor by slowly evaporation from at room.

### Refinement

The hydrogen atoms were placed in geometrically idealized positions with C*sp*^2^—H = 0.93Å and C*sp*^3^—H = 0.97 Å, and constrained to ride on their parent atoms with *U_iso_*(H) set to 1.2 × *U_eq_*(C).

### Crystal data

**Table:**

C_19_H_19_N_3_OS_2_	*Z* = 2
*M**_r_* = 369.49	*F*(000) = 388
Triclinic, *P*1	*D*_x_ = 1.402 Mg m^−^^3^
Hall symbol: -P 1	Mo *K*α radiation, λ = 0.71073 Å
*a* = 8.1484 (8) Å	Cell parameters from 2113 reflections
*b* = 9.3455 (9) Å	θ = 0.9–0.8°
*c* = 12.1834 (12) Å	µ = 0.32 mm^−^^1^
α = 73.668 (1)°	*T* = 298 K
β = 88.629 (1)°	Block, yellow
γ = 79.568 (1)°	0.30 × 0.23 × 0.18 mm
*V* = 875.26 (15) Å^3^	

### Data collection

**Table:**

Bruker SMART CCD area-detector diffractometer	3349 independent reflections
Radiation source: fine-focus sealed tube	2709 reflections with *I* > 2σ(*I*)
graphite	*R*_int_ = 0.016
ω scans	θ_max_ = 26.0°, θ_min_ = 1.7°
Absorption correction: multi-scan (*SADABS*; Sheldrick, 2000)	*h* = −10→7
*T*_min_ = 0.911, *T*_max_ = 0.945	*k* = −11→11
4828 measured reflections	*l* = −15→14

### Refinement

**Table:**

Refinement on *F*^2^	Primary atom site location: structure-invariant direct methods
Least-squares matrix: full	Secondary atom site location: difference Fourier map
*R*[*F*^2^ > 2σ(*F*^2^)] = 0.043	Hydrogen site location: inferred from neighbouring sites
*wR*(*F*^2^) = 0.119	H-atom parameters constrained
*S* = 1.06	*w* = 1/[σ^2^(*F*_o_^2^) + (0.0534*P*)^2^ + 0.3774*P*] where *P* = (*F*_o_^2^ + 2*F*_c_^2^)/3
3349 reflections	(Δ/σ)_max_ < 0.001
226 parameters	Δρ_max_ = 0.50 e Å^−^^3^
0 restraints	Δρ_min_ = −0.44 e Å^−^^3^

### Special details

**Table:**

Geometry. All e.s.d.'s (except the e.s.d. in the dihedral angle between two l.s. planes) are estimated using the full covariance matrix. The cell e.s.d.'s are taken into account individually in the estimation of e.s.d.'s in distances, angles and torsion angles; correlations between e.s.d.'s in cell parameters are only used when they are defined by crystal symmetry. An approximate (isotropic) treatment of cell e.s.d.'s is used for estimating e.s.d.'s involving l.s. planes.
Refinement. Refinement of *F*^2^ against ALL reflections. The weighted *R*-factor *wR* and goodness of fit *S* are based on *F*^2^, conventional *R*-factors *R* are based on *F*, with *F* set to zero for negative *F*^2^. The threshold expression of *F*^2^ > σ(*F*^2^) is used only for calculating *R*-factors(gt) *etc*. and is not relevant to the choice of reflections for refinement. *R*-factors based on *F*^2^ are statistically about twice as large as those based on *F*, and *R*- factors based on ALL data will be even larger.

### Fractional atomic coordinates and isotropic or equivalent isotropic displacement parameters (Å^2^)

**Table:**

	*x*	*y*	*z*	*U*_iso_*/*U*_eq_	
S1	0.47598 (8)	0.15501 (7)	0.76959 (5)	0.04843 (19)	
S2	0.06845 (11)	−0.09908 (9)	0.87874 (6)	0.0725 (3)	
N1	0.5900 (2)	0.2755 (2)	0.55975 (14)	0.0395 (4)	
N3	0.4362 (2)	0.27799 (19)	0.39525 (13)	0.0364 (4)	
N2	0.6791 (2)	0.3883 (2)	0.38313 (14)	0.0370 (4)	
O1	0.2152 (2)	0.1535 (2)	0.40870 (14)	0.0604 (5)	
C7	0.3484 (3)	0.1486 (2)	0.58029 (17)	0.0374 (5)	
C5	0.4765 (3)	0.1994 (2)	0.62133 (17)	0.0383 (5)	
C13	0.8080 (3)	0.4343 (3)	0.44164 (18)	0.0409 (5)	
H13A	0.8767	0.3469	0.4935	0.049*	
H13B	0.7579	0.5036	0.4844	0.049*	
C9	0.5685 (3)	0.3119 (2)	0.44757 (16)	0.0346 (4)	
C10	0.7206 (3)	0.4014 (3)	0.26241 (17)	0.0419 (5)	
H10A	0.6474	0.4861	0.2112	0.050*	
H10B	0.7122	0.3092	0.2429	0.050*	
C14	0.3260 (3)	0.5119 (2)	0.24945 (17)	0.0400 (5)	
H14	0.3266	0.5642	0.3041	0.048*	
C15	0.3794 (3)	0.2783 (3)	0.1980 (2)	0.0505 (6)	
H15	0.4169	0.1740	0.2178	0.061*	
C6	0.2442 (3)	0.0770 (2)	0.66839 (18)	0.0407 (5)	
C8	0.3242 (3)	0.1861 (2)	0.45921 (18)	0.0404 (5)	
C3	0.0878 (3)	0.0243 (3)	0.6458 (2)	0.0483 (6)	
H3A	0.1178	−0.0747	0.6326	0.058*	
H3B	0.0316	0.0940	0.5769	0.058*	
C4	0.2990 (3)	0.0722 (3)	0.77344 (19)	0.0460 (5)	
C11	0.8993 (3)	0.4273 (3)	0.25634 (19)	0.0491 (6)	
H11A	0.9223	0.4878	0.1807	0.059*	
H11B	0.9775	0.3318	0.2752	0.059*	
C12	0.9097 (3)	0.5119 (3)	0.34500 (19)	0.0479 (6)	
H12A	1.0244	0.5019	0.3700	0.057*	
H12B	0.8620	0.6186	0.3150	0.057*	
C18	0.2692 (3)	0.5888 (3)	0.13848 (19)	0.0523 (6)	
H18	0.2326	0.6933	0.1183	0.063*	
C1	−0.0312 (3)	0.0142 (3)	0.7442 (2)	0.0656 (7)	
H1A	−0.1244	−0.0294	0.7287	0.079*	
H1B	−0.0750	0.1156	0.7496	0.079*	
C17	0.2670 (4)	0.5117 (4)	0.0587 (2)	0.0652 (8)	
H17	0.2289	0.5640	−0.0157	0.078*	
C2	0.2226 (4)	0.0138 (3)	0.8875 (2)	0.0639 (7)	
H2A	0.1707	0.0990	0.9146	0.077*	
H2B	0.3099	−0.0470	0.9425	0.077*	
C16	0.3206 (4)	0.3571 (4)	0.0874 (2)	0.0660 (8)	
H16	0.3174	0.3053	0.0328	0.079*	
C19	0.3813 (2)	0.3578 (2)	0.27804 (16)	0.0362 (5)	

### Atomic displacement parameters (Å^2^)

**Table:**

	*U*^11^	*U*^22^	*U*^33^	*U*^12^	*U*^13^	*U*^23^
S1	0.0565 (4)	0.0596 (4)	0.0322 (3)	−0.0235 (3)	0.0016 (2)	−0.0098 (2)
S2	0.0983 (6)	0.0831 (5)	0.0519 (4)	−0.0533 (5)	0.0301 (4)	−0.0233 (4)
N1	0.0401 (10)	0.0474 (10)	0.0320 (9)	−0.0142 (8)	−0.0005 (7)	−0.0089 (8)
N3	0.0368 (9)	0.0427 (9)	0.0302 (8)	−0.0127 (8)	−0.0029 (7)	−0.0073 (7)
N2	0.0354 (9)	0.0487 (10)	0.0308 (8)	−0.0153 (8)	0.0017 (7)	−0.0128 (7)
O1	0.0633 (11)	0.0780 (12)	0.0444 (9)	−0.0425 (10)	−0.0097 (8)	−0.0050 (8)
C7	0.0400 (11)	0.0339 (10)	0.0361 (11)	−0.0085 (9)	−0.0015 (9)	−0.0053 (8)
C5	0.0420 (11)	0.0402 (11)	0.0322 (10)	−0.0102 (9)	0.0008 (9)	−0.0078 (9)
C13	0.0378 (11)	0.0512 (13)	0.0377 (11)	−0.0149 (10)	−0.0038 (9)	−0.0143 (10)
C9	0.0350 (10)	0.0375 (10)	0.0321 (10)	−0.0082 (9)	−0.0006 (8)	−0.0102 (8)
C10	0.0371 (11)	0.0597 (14)	0.0324 (11)	−0.0131 (10)	0.0027 (9)	−0.0158 (10)
C14	0.0365 (11)	0.0520 (13)	0.0324 (10)	−0.0137 (10)	0.0018 (8)	−0.0100 (9)
C15	0.0524 (14)	0.0603 (15)	0.0471 (13)	−0.0160 (12)	0.0031 (11)	−0.0249 (11)
C6	0.0433 (12)	0.0350 (11)	0.0416 (12)	−0.0100 (9)	0.0012 (9)	−0.0055 (9)
C8	0.0412 (12)	0.0398 (11)	0.0405 (11)	−0.0130 (10)	−0.0034 (9)	−0.0080 (9)
C3	0.0481 (13)	0.0464 (13)	0.0484 (13)	−0.0182 (11)	0.0020 (10)	−0.0042 (10)
C4	0.0514 (13)	0.0464 (12)	0.0414 (12)	−0.0172 (11)	0.0070 (10)	−0.0097 (10)
C11	0.0388 (12)	0.0715 (16)	0.0441 (12)	−0.0180 (11)	0.0081 (10)	−0.0235 (12)
C12	0.0403 (12)	0.0599 (14)	0.0498 (13)	−0.0198 (11)	0.0049 (10)	−0.0189 (11)
C18	0.0510 (14)	0.0613 (15)	0.0376 (12)	−0.0139 (12)	−0.0022 (10)	−0.0002 (11)
C1	0.0525 (15)	0.0728 (18)	0.083 (2)	−0.0252 (14)	0.0168 (14)	−0.0334 (16)
C17	0.0686 (18)	0.095 (2)	0.0297 (12)	−0.0255 (16)	−0.0049 (11)	−0.0070 (13)
C2	0.0761 (19)	0.0790 (18)	0.0458 (14)	−0.0381 (16)	0.0188 (13)	−0.0187 (13)
C16	0.0736 (19)	0.099 (2)	0.0394 (13)	−0.0287 (17)	0.0014 (12)	−0.0347 (14)
C19	0.0317 (10)	0.0490 (12)	0.0298 (10)	−0.0128 (9)	−0.0002 (8)	−0.0107 (9)

### Geometric parameters (Å, °)

**Table:**

S1—C5	1.736 (2)	C14—H14	0.9300
S1—C4	1.750 (2)	C15—C19	1.385 (3)
S2—C1	1.801 (3)	C15—C16	1.390 (4)
S2—C2	1.803 (3)	C15—H15	0.9300
N1—C9	1.320 (3)	C6—C4	1.352 (3)
N1—C5	1.352 (3)	C6—C3	1.504 (3)
N3—C9	1.392 (3)	C3—C1	1.517 (3)
N3—C8	1.432 (3)	C3—H3A	0.9700
N3—C19	1.451 (2)	C3—H3B	0.9700
N2—C9	1.350 (3)	C4—C2	1.507 (3)
N2—C13	1.473 (2)	C11—C12	1.519 (3)
N2—C10	1.477 (2)	C11—H11A	0.9700
O1—C8	1.220 (3)	C11—H11B	0.9700
C7—C5	1.379 (3)	C12—H12A	0.9700
C7—C8	1.427 (3)	C12—H12B	0.9700
C7—C6	1.442 (3)	C18—C17	1.366 (4)
C13—C12	1.513 (3)	C18—H18	0.9300
C13—H13A	0.9700	C1—H1A	0.9700
C13—H13B	0.9700	C1—H1B	0.9700
C10—C11	1.516 (3)	C17—C16	1.377 (4)
C10—H10A	0.9700	C17—H17	0.9300
C10—H10B	0.9700	C2—H2A	0.9700
C14—C19	1.375 (3)	C2—H2B	0.9700
C14—C18	1.386 (3)	C16—H16	0.9300
			
C5—S1—C4	91.1 (1)	C1—C3—H3A	109.1
C1—S2—C2	98.8 (1)	C6—C3—H3B	109.1
C9—N1—C5	115.5 (2)	C1—C3—H3B	109.1
C9—N3—C8	122.0 (2)	H3A—C3—H3B	107.9
C9—N3—C19	122.1 (2)	C6—C4—C2	128.5 (2)
C8—N3—C19	114.7 (2)	C6—C4—S1	113.0 (2)
C9—N2—C13	118.4 (2)	C2—C4—S1	118.5 (2)
C9—N2—C10	128.8 (2)	C10—C11—C12	103.6 (2)
C13—N2—C10	110.9 (2)	C10—C11—H11A	111.0
C5—C7—C8	117.7 (2)	C12—C11—H11A	111.0
C5—C7—C6	113.8 (2)	C10—C11—H11B	111.0
C8—C7—C6	128.2 (2)	C12—C11—H11B	111.0
N1—C5—C7	127.4 (2)	H11A—C11—H11B	109.0
N1—C5—S1	121.8 (2)	C13—C12—C11	103.2 (2)
C7—C5—S1	110.8 (2)	C13—C12—H12A	111.1
N2—C13—C12	103.8 (2)	C11—C12—H12A	111.1
N2—C13—H13A	111.0	C13—C12—H12B	111.1
C12—C13—H13A	111.0	C11—C12—H12B	111.1
N2—C13—H13B	111.0	H12A—C12—H12B	109.1
C12—C13—H13B	111.0	C17—C18—C14	120.2 (2)
H13A—C13—H13B	109.0	C17—C18—H18	119.9
N1—C9—N2	117.2 (2)	C14—C18—H18	119.9
N1—C9—N3	122.8 (2)	C3—C1—S2	112.3 (2)
N2—C9—N3	120.0 (2)	C3—C1—H1A	109.2
N2—C10—C11	103.5 (2)	S2—C1—H1A	109.2
N2—C10—H10A	111.1	C3—C1—H1B	109.2
C11—C10—H10A	111.1	S2—C1—H1B	109.2
N2—C10—H10B	111.1	H1A—C1—H1B	107.9
C11—C10—H10B	111.1	C18—C17—C16	120.5 (2)
H10A—C10—H10B	109.0	C18—C17—H17	119.7
C19—C14—C18	119.4 (2)	C16—C17—H17	119.7
C19—C14—H14	120.3	C4—C2—S2	111.9 (2)
C18—C14—H14	120.3	C4—C2—H2A	109.2
C19—C15—C16	118.8 (2)	S2—C2—H2A	109.2
C19—C15—H15	120.6	C4—C2—H2B	109.2
C16—C15—H15	120.6	S2—C2—H2B	109.2
C4—C6—C7	111.3 (2)	H2A—C2—H2B	107.9
C4—C6—C3	124.3 (2)	C17—C16—C15	120.1 (2)
C7—C6—C3	124.3 (2)	C17—C16—H16	119.9
O1—C8—C7	126.3 (2)	C15—C16—H16	119.9
O1—C8—N3	119.5 (2)	C14—C19—C15	121.0 (2)
C7—C8—N3	114.1 (2)	C14—C19—N3	118.9 (2)
C6—C3—C1	112.4 (2)	C15—C19—N3	120.1 (2)
C6—C3—H3A	109.1		
			
C9—N1—C5—C7	4.1 (3)	C19—N3—C8—O1	16.0 (3)
C9—N1—C5—S1	−175.1 (2)	C9—N3—C8—C7	7.0 (3)
C8—C7—C5—N1	−3.7 (3)	C19—N3—C8—C7	−160.6 (2)
C6—C7—C5—N1	−177.4 (2)	C4—C6—C3—C1	19.0 (3)
C8—C7—C5—S1	175.5 (2)	C7—C6—C3—C1	−156.5 (2)
C6—C7—C5—S1	1.9 (2)	C7—C6—C4—C2	177.0 (2)
C4—S1—C5—N1	178.0 (2)	C3—C6—C4—C2	1.0 (4)
C4—S1—C5—C7	−1.3 (2)	C7—C6—C4—S1	0.6 (3)
C9—N2—C13—C12	178.6 (2)	C3—C6—C4—S1	−175.5 (2)
C10—N2—C13—C12	13.1 (2)	C5—S1—C4—C6	0.4 (2)
C5—N1—C9—N2	179.8 (2)	C5—S1—C4—C2	−176.4 (2)
C5—N1—C9—N3	1.5 (3)	N2—C10—C11—C12	−30.9 (2)
C13—N2—C9—N1	−4.0 (3)	N2—C13—C12—C11	−31.9 (2)
C10—N2—C9—N1	158.6 (2)	C10—C11—C12—C13	39.2 (2)
C13—N2—C9—N3	174.4 (2)	C19—C14—C18—C17	0.6 (3)
C10—N2—C9—N3	−23.0 (3)	C6—C3—C1—S2	−53.3 (3)
C8—N3—C9—N1	−7.1 (3)	C2—S2—C1—C3	62.2 (2)
C19—N3—C9—N1	159.5 (2)	C14—C18—C17—C16	0.1 (4)
C8—N3—C9—N2	174.5 (2)	C6—C4—C2—S2	14.9 (4)
C19—N3—C9—N2	−18.8 (3)	S1—C4—C2—S2	−168.8 (2)
C9—N2—C10—C11	−152.5 (2)	C1—S2—C2—C4	−40.8 (2)
C13—N2—C10—C11	11.2 (2)	C18—C17—C16—C15	−0.7 (4)
C5—C7—C6—C4	−1.6 (3)	C19—C15—C16—C17	0.7 (4)
C8—C7—C6—C4	−174.4 (2)	C18—C14—C19—C15	−0.7 (3)
C5—C7—C6—C3	174.4 (2)	C18—C14—C19—N3	−178.4 (2)
C8—C7—C6—C3	1.6 (4)	C16—C15—C19—C14	0.1 (3)
C5—C7—C8—O1	−178.3 (2)	C16—C15—C19—N3	177.7 (2)
C6—C7—C8—O1	−5.7 (4)	C9—N3—C19—C14	−60.2 (3)
C5—C7—C8—N3	−1.9 (3)	C8—N3—C19—C14	107.4 (2)
C6—C7—C8—N3	170.6 (2)	C9—N3—C19—C15	122.1 (2)
C9—N3—C8—O1	−176.4 (2)	C8—N3—C19—C15	−70.3 (2)

### Hydrogen-bond geometry (Å, °)

**Table:**

*D*—H···*A*	*D*—H	H···*A*	*D*···*A*	*D*—H···*A*
C11—H11B···O1^i^	0.97	2.58	3.434 (3)	148.
C1—H1A···O1^ii^	0.97	2.48	3.287 (3)	140.
C3—H3B···O1	0.97	2.50	3.038 (3)	115.

Symmetry codes: (i) *x*+1, *y*, *z*; (ii) −*x*, −*y*, −*z*+1.
